# Nocturnal autonomic activity in athletes with regular versus prolonged return to sport after sport-related concussion

**DOI:** 10.1038/s41598-026-43546-0

**Published:** 2026-03-26

**Authors:** Anne Carina Delling-Brett, Rasmus Jakobsmeyer, Jessica Coenen, Claus Reinsberger

**Affiliations:** 1 Institute of Sports Medicine, Department of Exercise and Health, Paderborn University, Warburger Straße 100, 33098 Paderborn, Germany; 2 Division of Sports Neurology and Neurosciences, Department of Neurology, Mass General Brigham, Boston, MA USA; 3 Neuromotor Behavior and Exercise, Institute of Sport and Exercise Sciences, University of Muenster, Wilhelm-Schickard-Straße 8, 48149 Münster, Germany

**Keywords:** Concussion, sport, autonomic nervous system, sleep, heart rate variability, electrodermal activity, Neurology, Neuroscience, Physiology

## Abstract

Sport-related concussion (SRC) is a multifaceted brain injury linked to altered autonomic activity, which may persist and contribute to prolonged recovery and persisting post-concussion symptoms (PPCS). This exploratory cross-sectional study addressed whether athletes with prolonged recovery show altered nocturnal autonomic activity after SRC, offering potential insights into PPCS and biomarkers. Nocturnal autonomic activity and concussion symptoms were evaluated and compared between 17 SRC athletes and 17 matched control athletes. SRC athletes were classified by individual return to sport (RTS) into regular RTS (< 28 days, *n* = 10) and prolonged RTS (≥ 28 days, *n* = 7). Nocturnal autonomic measures were collected during and post RTS (> 3 weeks) using a multimodal wearable device. Outcomes included heart rate (HR), HR variability (RMSSD), and electrodermal activity (EDA). No group differences in concussion symptoms, HR, RMSSD, and EDA were found during RTS. Post RTS, prolonged RTS athletes displayed significantly lower nocturnal RMSSD (*p* = 0.035, *r* = 0.612) than regular RTS athletes and controls. Additionally, prolonged RTS athletes exhibited fewer phasic EDA (sleep storms) compared to regular RTS athletes. Prolonged SRC recovery might be associated with reduced nocturnal parasympathetic (RMSSD) and phasic sympathetic (EDA) activity, after clinical symptom resolution. Further research is needed to explore whether this reflects insufficient physiological recovery or deconditioning.

**Supplementary Information:**

The online version contains supplementary material available at 10.1038/s41598-026-43546-0.

## Introduction

Sport-related concussion (SRC) is defined as the mildest subtype of mild traumatic brain injury (mTBI) in the context of sports due to a direct or indirect transmission of biomechanical forces to the head, resulting in a variety of symptoms^1^. Most athletes recover clinically (defined by full symptom resolution) within approximately four weeks^2–5^ under established return to sport (RTS) protocols^1,2^. Yet 10–30% of athletes experience persistent post-concussion symptoms (PPCS) associated with prolonged RTS^5,6^. Initial symptom number and severity may serve as predictors of prolonged recovery^2^, and initial sleep-related symptoms (“trouble falling asleep”, “fatigue”, and “drowsiness”) were linked to an increased risk of persisting symptoms^3^.

Autonomic nervous system (ANS) dysfunction may be a prominent feature of both acute SRC and PPCS^4,5^. Consequently, assessments of the ANS have been added to recent clinical guidelines of SRC^1,5^. The ANS unconsciously maintains homeostasis in the body through its parasympathetic and sympathetic branches^6^. It has been hypothesized that an imbalance in their activity may reflect pathophysiological mechanisms after SRC, characterized by increased sympathetic and reduced parasympathetic activity^7^. Previous research has predominantly focused on cardiac ANS dysfunction, assessing measures of heart rate variability (HRV) or heart rate (HR), which are easily obtainable due to their non-invasive and cost-effective nature^4^. HRV parameters primarily reflect parasympathetic (such as the root mean square of successive interbeat interval differences (RMSSD)) or a combination of parasympathetic and sympathetic activity, making observations on pure sympathetic activity challenging^8^.

Electrodermal activity (EDA), which reflects sweat gland activity modulated solely by sympathetic innervation, offers a more direct measure of sympathetic activity and may provide exploratory insights into potential ANS dysfunction after SRC^9^. It is analyzed by assessing a tonic component, representing the slowly changing baseline (EDA level), and a phasic component, characterized by rapid transient changes (EDA responses, EDRs), overlaying the tonic activity^9^. Intraindividual differences in EDA during the day are associated with varying levels of arousal (physiological and psychological activation of the organism through central nervous activation), with higher sympathetic nervous system arousal leading to increased EDA^10^. During deep sleep, particularly in the first half of the night, high-frequency bursts of EDRs termed sympathetic “sleep storms” have been observed^11^. They are hypothesized to play a role in cognitive functions, such as memory consolidation^12^, and might therefore be considered part of normal sleep physiology rather than a pathological phenomenon. At the same time, cardiac parasympathetic activity is supposed to be predominant during this sleep phase, accompanied by reduced cardiac sympathetic modulation^13^.

Sleep plays a vital role in brain recovery after injury, particularly through sleep-dependent synaptic plasticity^14^. However, 30% to 70% of athletes report impaired sleep after SRC^15^, affecting the duration of recovery^16^. Assessing ANS activity during sleep may provide valuable insights into the physiological processes underlying (neurophysiological) recovery^13^. In conditions such as insomnia and hyperarousal, ANS dysfunction has been proposed as a possible underlying mechanism^17^, yet its role in sleep problems after SRC remains unexplored. Additionally, nocturnal recordings offer a non-obtrusive and standardized setting for ANS measurements, minimizing psychological and environmental influences^18^, with a positive impact on data quality compared to daytime measurements^19^ and high participant compliance^20^.

ANS dysfunction is a typical feature of TBI and may even exceed clinical recovery after SRC^21^. ANS assessments might therefore allow the evaluation of (patho-) physiological alterations that may persist beyond symptom resolution. Thus, this exploratory study aimed to analyze indicators of nocturnal autonomic activity in athletes with regular versus prolonged RTS, both during and after RTS, using a multimodal wearable device. It was hypothesized that athletes with prolonged RTS may initially present a greater number and severity of concussion symptoms. Furthermore, it was assumed that prolonged RTS athletes in comparison to athletes with regular RTS and healthy controls, would potentially exhibit persistent alterations in nocturnal autonomic activity, particularly shown by reduced parasympathetic modulation. Therefore, RMSSD and HR were used as primary outcomes, and EDA as a secondary outcome, to assess nocturnal autonomic activity.

## Results

### Subjects and concussion symptoms

Seventeen adult elite athletes diagnosed with SRC were enrolled, and nocturnal autonomic activity was assessed during (TP1) and after (three weeks post RTS, TP2) individual RTS protocols. Based on the clinical course, athletes were retrospectively divided into a prolonged RTS (pRTS; ≥ 28 days until RTS; *n* = 7) and a regular RTS group (rRTS; < 28 days until RTS; *n* = 10). Nocturnal measures of control subjects (*n* = 17), matched for sex, age, height, weight, sports, and expertise level, were used for comparison.

**Table 1:** Subject characteristics presented as mean (± SD).

	Controls (*n* = 17)	rRTS athletes (*n* = 10)	pRTS athletes (*n* = 7)	*p*-value (effect size)
Sex (*n*)	m = 14, f = 3	m = 9, f = 1	m = 5, f = 2	
Age (yrs.)	23 (± 5)	22 (± 3)	24 (± 7)	0.961_a_
Height (m)	1.84 (± 0.10)	1.83 (± 0.06)	1.85 (± 0.14)	0.967_a_
Weight (kg)	81 (± 14)	80 (± 9)	83 (± 18)	0.834_a_
BMI (kg/m^2^)	24 (± 2)	24 (± 2)	24 (± 3)	0.976_a_
Previous concussions (n)	1 (± 1)	1 (± 1)	1 (± 2)	0.647_a_
RTS in days	/	13 (± 4)	74 (± 53)	0.023_b_* (0.733)
Days between injury and inclusion	/	3 (± 2)	25 (± 31)	0.009_d_* (0.609)
Days between injury and last night recording (TP2)	/	44 (± 29)	96 (± 52)	0.002_d_* (0.701)
Sport (n)	Soccer (7) Basketball (4) Am. Football (4) Handball (1) Modern Pentathlon (1)	Soccer (7) Am. Football (2) Basketball (1)	Basketball (2) Handball (2) Soccer (1) Ice Hockey (1) Modern Pentathlon (1)	
Medical history (n)	Migraine (2)	Thyroid dysfunction (1) Depression (1)	Migraine (2) Depression (1) Learning disability (1) Thyroid dysfunction (1)	0.069_c_

RTS = return to sport; rRTS = regular RTS; pRTS = prolonged RTS; m = male; f = female, BMI = body mass index; * *p* < 0.05 significant group differences (_a_ = Kruskal-Wallis-test, _b_ = t-Test, _c_ = Fisher’s exact test, _d_ = Mann-Whitney U test).

There were no significant differences between groups in demographics or number of previous concussions or medical history (Table 1). Duration of RTS was significantly longer in the pRTS (74 ± 53 days) than in the rRTS group (13 ± 4 days, t_(6)_ = -3.009; *p* = 0.023; *r* = 0.733). Significant group differences in symptoms, assessed by the symptom checklist of the “Sport Concussion Assessment Tool 5” (SCAT5), are presented in Table 2. Clinical assessments were obtained at non-equivalent time points and occurred significantly later in the pRTS group (25 ± 31 days post-injury) compared with the rRTS athletes (3 ± 2 days, U = 9.500, z = -2.512, *p* = 0.009, *r* = -0.609). Several symptoms were significantly more often reported in both SRC groups compared to controls such as “headache” (pRTS athletes: Median (Mdn) = 3, *p* = 0.004, z = 3,234, *r* = 0.660; rRTS athletes: Mdn = 1.5, *p* = 0.029, z = 2.591, *r* = 0.499), “pressure in head” (pRTS athletes: Mdn = 3, *p* = 0.001, z = 3,680, *r* = 0.751; rRTS athletes: Mdn = 1.5, *p* = 0.006, z = 3.074, *r* = 0.592), and “fatigue or low energy” (pRTS athletes: Mdn = 3, *p* = 0.004, z = 3.219, *r* = 0.657; rRTS athletes: Mdn = 2, *p* = 0.031, z = 2.569, *r* = 0.495). rRTS athletes presented significantly higher values for “drowsiness” (Mdn) = 1.5, z = 2.476, *p* = 0.040, *r* = 0.476) compared to controls (Mdn = 0). pRTS athletes reported significantly more “dizziness” (Mdn = 1, z = 2.549, *p* = 0.032, *r* = 0.520), “balance problems” (Mdn = 0, z = 2.645, *p* = 0.025, *r* = 0.539) and “feeling slowed down” (Mdn = 1, z = 3.208, *p* = 0.004, *r* = 0.654) compared to controls (Mdn for these symptoms = 0). Symptom domain clustering is available in Supplementary Table S1 online.

**Table 2:** Concussion symptoms presented as median (± SD).

	Controls (*n* = 17)	rRTS athletes (*n* = 10)	pRTS athletes (*n* = 7)
Symptom number	1.00 (± 4.43)	6.50* (± 5.24)	12.00* (± 4.23)
Symptom severity	1.00 (± 6.39)	11.50* (± 15.02)	28.00* (± 15.10)
Headache	0.00 (± 0.49)	1.50* (± 1.43)	3.00* (± 1.98)
Pressure in head	0.00 (± 0.53)	1.50* (± 1.25)	3.00* (± 1.62)
Neck pain	0.00 (± 0.39)	1.50 (± 1.71)	1.00 (± 1.13)
Dizziness	0.00 (± 0.49)	0.00 (± 0.70)	1.00* (± 1.07)
Balance problems	0.00 (± 0.00)	0.00 (± 0.97)	0.00* (± 1.22)
Feeling slowed down	0.00 (± 0.24)	0.00 (± 1.41)	1.00* (± 1.70)
Don’t feel right	0.00 (± 0.24)	1.00* (± 1.27)	3.00* (± 2.14)
Difficulty concentrating	0.00 (± 0.24)	1.00* (± 1.65)	3.00* (± 1.00)
Difficulty remembering	0.00 (± 0.53)	0.50 (± 1.25)	1.00 (± 1.38)
Fatigue or low energy	0.00 (± 0.72)	2.00* (± 1.63)	3.00* (± 1.86)
Drowsiness	0.00 (± 0.56)	1.50* (± 1.84)	0.00 (± 0.98)

RTS = return to sport; rRTS = regular RTS; pRTS = prolonged RTS; * *p* < 0.05 different to controls (Kruskal-Wallis-test with post hoc test).

### Nocturnal autonomic activity

**Fig. 1:**
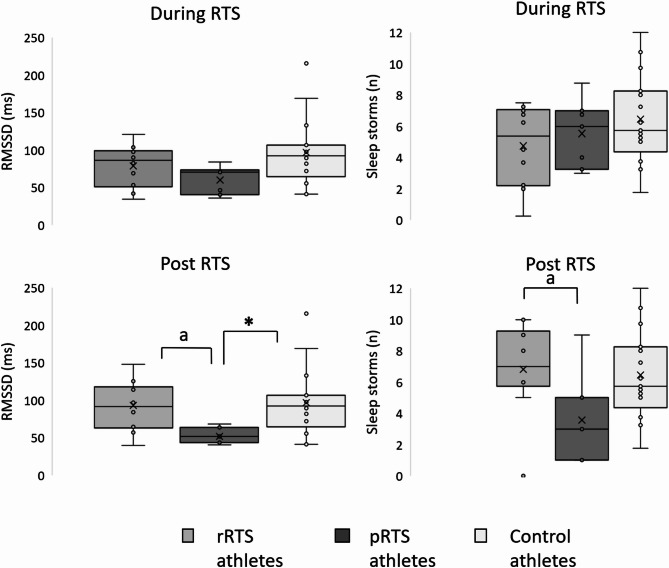
Nocturnal RMSSD and sleep storms during and post return to sport (RTS) for regular (rRTS), prolonged (pRTS), and control athletes. * = significant differences between pRTS and control athletes; a = significant differences between rRTS and pRTS athletes (applying Kruskal-Wallis Test with Post-hoc tests (Dunn-Bonferroni-Tests)).

Indicators of sleep and results of nocturnal ANS activity are displayed in Table 3; Fig. 1. During RTS (TP1), nocturnal RMSSD as the primary outcome did not show differences between pRTS (Mdn = 70.88ms), rRTS (Mdn = 86.31ms), and control athletes (Mdn = 92.71ms, *p* = 0.051, d = 0.765). Post-hoc analysis revealed a significantly reduced nocturnal RMSSD (z = -2.433, *p* = 0.045, *r* = -0.496) in pRTS athletes compared to controls but not to rRTS athletes (z = 1.426, *p* = 0.461, *r* = 0.345). There were no differences between all groups in the other nocturnal parameters at TP1.

After completion of RTS (TP2), nocturnal RMSSD of pRTS athletes (Mdn = 51.77ms) was significantly lower compared to rRTS athletes (Mdn = 91.43ms, z = 2.524, *p* = 0.035, *r* = 0.612) and to controls (Mdn = 92.71ms, z = -2.747, *p* = 0.018, *r* = -0.560). The percentage change of RMSSD from during to post RTS differed significantly between rRTS (Mdn = + 8.16%) and pRTS athletes (Mdn = -11.88%, U = 12.00, z = -2.245, *p* = 0.025, *r* = -0.544).

To account for interindividual differences, an ANCOVA was conducted comparing post RTS (TP2) RMSSD between rRTS and pRTS athletes while controlling for RMSSD during RTS (TP1). This analysis revealed a significant main effect of group (*F*(1,14) = 7.75, *p* = 0.015, partial η² = 0.36), indicating lower RMSSD values at TP2 in pRTS athletes. The covariate was also significant (*F*(1,14) = 22.06, *p* = 0.001, partial η² = 0.61), demonstrating that higher RMSSD at TP1 predicted higher RMSSD at TP2.

At TP2, pRTS athletes exhibited reduced phasic EDA compared to rRTS athletes, displayed by fewer sleep storms (Mdn = 3 vs. Mdn = 7, *p* = 0.046, *r* = 0.588). The percentage change of sleep storms increased significantly from TP1 to TP2 in rRTS (Mdn = + 33.33%) but decreased in pRTS athletes (Mdn = -25.00%, U = 9.00, z = -2.539, *p* = 0.009, *r* = -0.616). Using ANCOVA, these exploratory observations were controlled for sleep storms at TP1, revealing a significant main effect of group on sleep storm frequency at TP2 (*F*(1,14) = 9.83, *p* = 0.007, partial η² = 0.41), with pRTS athletes exhibiting fewer sleep storms at TP2 compared to rRTS athletes.

**Table 3:** Nocturnal ANS data presented as median (± SD).

	Controls (*n* = 17)	rRTS athletes (*n* = 10)			pRTS athletes (*n* = 7)		
		during RTS	post RTS	Δ diff (%)	during RTS	post RTS	Δ diff (%)
HR(V) parameters							
HR (bpm)	57.28 (± 7.69)	50.34 (± 7.74)	47.88 (± 7.08)	-1.17	58.09 (± 6.30)	59.26 (± 5.56)	5.57
CV HR (%)	6.22	6.19	/		6.12	/	
RMSSD (ms)	92.71 (± 44.20)	86.31 (± 28.12)	91.43 (± 33.62)	22.08°^b^	70.88 (± 18.86)	51.77*°^a^ (± 10.58)	-11.88
CV RMSSD (%)	25.15	22.44	/		23.82	/	
EDA parameters							
CDA.Tonic (µS)	3.47 (± 3.05)	1.95 (± 2.42)	3.13 (± 3.92)	5.12	3.28 (± 3.05)	1.46 (± 6.34)	-7.96
CV CDA.Tonic (%)	69.00	58.00	/		39.00	/	
CDA.SCR (µS)	0.023 (± 0.02)	0.011 (± 0.02)	0.016 (± 0.02)	59.62	0.014 (± 0.02)	0.010 (± 0.01)	-30.10
CV CDA.SCR (%)	57.00	72.50	/		34.00	/	
Sleep storms (n)	5.75 (± 2.78)	5.38 (± 2.59)	7.00 (± 2.97)	33.33°^b^	6.00 (± 2.17)	3.00°^a^ (± 2.76)	-25.00
CV Sleep storms (%)	48.00	58.50	/		30.00	/	

RTS = return to sport; rRTS = regular RTS; pRTS = prolonged RTS; CV = coefficient of variation; Δ diff = % difference from during to post RTS; * *p* < 0.05 different to controls (Kruskal-Wallis-test with post hoc test); ° *p* < 0.05 different between RTS groups (_a_ = Kruskal-Wallis-test with post hoc test, _b_ = Mann Whitney U-test).

## Discussion

This explorative study aimed to investigate nocturnal ANS activity and concussion symptoms in athletes with regular versus prolonged RTS after SRC. Athletes with prolonged RTS exhibited reduced cardiac parasympathetic activity compared to controls and athletes with a regular RTS, whereas electrodermal phasic sympathetic activity was diminished only to rRTS athletes. These findings may indicate persistent alterations in autonomic regulation in the pRTS group and extend previous daytime HRV findings to nocturnal conditions.

At the initial assessment, pRTS athletes reported a greater number and severity of symptoms compared to rRTS athletes (Table 2), albeit not reaching statistical significance. The heterogeneity of time after SRC at the initial presentation and study enrollment (see Table 1) may contribute to this finding, as concussion symptoms vary over time^22^ and are likely to decrease within the first two weeks after SRC^2,23^. Notably, rRTS athletes reported more “drowsiness” compared to matched controls (*p* = 0.040, *r* = 0.476), similar to an investigation of a sample of collegiate athletes assessing symptoms within 72 h after concussion^24^. In children after non-sport-related mTBI, drowsiness and the prevalence of sleep disturbances showed decrements with increasing time since injury^25^ which might explain why only rRTS revealed significantly more drowsiness. However, pRTS athletes reported more “dizziness”, “balance problems”, and “feeling slowed down” than controls, which aligns with previous work linking vestibulo-ocular and cognitive-related symptoms^26^ to a higher likelihood of PPCS^27–29^. Continuous symptom monitoring and individual comparisons to baseline values are necessary to validly attribute symptoms to SRC injury and effectively monitor symptom development during RTS in future studies.

Reduced nocturnal parasympathetic activity during RTS in the pRTS group approached significance (*p* = 0.051, d = 0.765) but became significant (*p* = 0.035, *r* = 0.612) post RTS (> 21 days after completion of RTS), suggesting sustained parasympathetic withdrawal even after medical clearance^11,32^. Importantly, when accounting for interindividual differences in autonomic activity during the RTS phase, group differences in post RTS RMSSD remained significant. Hutchison et al. (2017) similarly reported a lower parasympathetic activity (high-frequency band) at rest in concussed athletes (*n* = 26) compared to matched control athletes one week after their RTS^30^. Research in more severe types of TBI suggests that autonomic disturbances may scale with injury severity^31^, supporting the observation that reduced RMSSD was primarily evident in pRTS athletes. On the one hand, reduced cardiac parasympathetic activity may reflect the impact of SRC on brain regions involved in central autonomic regulation and might therefore serve as an indicator of ongoing SRC-related recovery processes^4^. On the other hand, decreased nocturnal RMSSD post RTS may also be explained by a reduction in training, potentially leading to deconditioning and subsequently reduced parasympathetic activity^32^. Without data on training and exercise behavior of those patients, this aspect remains speculative, yet possible. Moreover, nocturnal autonomic indices vary during different sleep stages^13^. Therefore, changes in sleep and sleep architecture following SRC^33^ may also contribute to changes in nocturnal RMSSD and should be addressed in future research.

In summary, reduced nocturnal cardiac parasympathetic activity could result from a more predominant dysfunction of the ANS and/or a reflection of insufficient physiological recovery, but also from changes in training load and sleep^5^. Obtaining baseline values of ANS activity and training status, as well as continuously analyzing nocturnal RMSSD after RTS in athletes, may help elucidate this effect in more detail. Additionally, expanding nocturnal autonomic activity to clinical RTS and return to performance, where the athlete can compete at the pre-injury level^34^ should be considered.

Current reviews on resting HRV after SRC^35^ and mTBI^4^ revealed inconsistent results. Approximately half of the included studies described significant differences between SRC and control subjects^4,35^. Methodological heterogeneity across studies, including differences in ANS measurement protocols, reported outcomes, study designs, participant characteristics, and variations in injury severity and timing of assessments, contributed to these inconsistent findings. As ANS activity is highly susceptible to environmental and psychological influences, recordings during sleep may offer a way to restrict external stimuli, allow for standardized measurement conditioning in a home-based setting^13,36^, and may thereby more accurately capture autonomic recovery processes following SRC than daytime laboratory assessments.

Sleep plays an essential role in physiological and cognitive recovery processes after concussion. Sleep-related symptoms (“trouble falling asleep”, “fatigue”, and “drowsiness”) have been associated with prolonged recovery, PPCS, and cognitive impairments, particularly in memory function^3^. Increased “fatigue” was reported in both SCR groups compared to control athletes. Fatigue is considered a key symptom of ANS dysfunction, which may also be linked to sleep-related problems. Individuals experiencing fatigue commonly present with increased sympathetic activity and reduced parasympathetic modulation, indicating an imbalance in autonomic regulation^37^. In addition, increased arousal has been considered a possible physiological mechanism associated with sleep problems in mTBI patients^21,33^ and SRC athletes^38^. EDA, as a candidate marker of sympathetic arousal, revealed that pRTS athletes exhibited significantly fewer sleep storms (*p* = 0.046, *r* = 0.588) compared to rRTS athletes post RTS. This group difference persisted after controlling for interindividual differences in sleep storm frequency during the RTS phase. Although nocturnal EDRs have been hypothesized to contribute to memory consolidation^45^, the functional significance of altered sleep storm frequency remains to be determined. Moreover, both EDRs and sleep storms showed high intraindividual variability^39^. Therefore, findings from a single night post RTS^23^ should be interpreted with caution. A high coefficient of variation was estimated for all EDA outcomes (Table 3), which is consistent with previous research and challenges statistical group comparisons^12^. Longitudinal investigations targeting intraindividual developments are more appropriate to examine individual changes in sleep physiology and arousal after SRC, possibly even in combination with polysomnography.

The current explorative study has several limitations that need to be considered. Both samples of elite athletes, with 7 athletes in the prolonged RTS and 10 athletes in the regular RTS group, are small. This limits statistical power and increases susceptibility to overfitting and false-positive findings, particularly given the high intra- and interindividual variability of ANS activity. Accordingly, the results should be interpreted as hypothesis-generating rather than confirmatory. The study sample consisted mostly of adult elite athletes, though findings may not be generalized to youth athletes or non-athlete populations with SRC. In addition, the low number of female participants (3 SRC and 3 control athletes) prevented assessment of sex-specific effects and may have confounded results, as females generally exhibit higher resting cardiac parasympathetic activity than men^40^. Concussion symptoms were collected only once at the inclusion of subjects into the study. The SCAT5 and ANS data collection was applied significantly (*p* = 0.009, *r* = -0.609) longer after the trauma in pRTS athletes, which might have diminished potential differences in symptom reporting between the SRC groups. As training during and post RTS was not monitored, it was not possible to analyze the impact of physical deconditioning on parasympathetic activity in pRTS athletes. Furthermore, time in bed was approximated on accelerometry data, which is a good approximation but might not be equal to sleep. Accelerometry data has been shown to have a high correlation with the gold standard for capturing sleep (polysomnography), particularly when assessing sleep time^41^. In our study, the first 4 h-period (except for sleep storms) was analyzed to standardize heterogeneous sleep durations and investigate primary deep sleep stages^42^, which can not be verified without performing polysomnography. The presence of different sleep stages in the analyzed data and their potential to contribute to additional variability in the ANS data cannot, therefore, be evaluated.

While no differences in concussion symptoms and ANS activity during RTS were found between rRTS and pRTS athletes, nocturnal cardiac parasympathetic and phasic sympathetic activity were, despite the limitations mentioned above, significantly reduced in pRTS compared to rRTS athletes after finalizing RTS. It remains to be elucidated whether these differences are a cause (indicating insufficient recovery after SRC and more severe functional injury or dysautonomia) or a consequence (e.g., physical deconditioning, changes in sleep physiology) of prolonged RTS. Still, these findings support the potential use of nocturnal HRV and EDA as non-invasive research tools for assessing physiological recovery after SRC, warranting validation in larger, longitudinal cohorts with baseline values.

## Methods

This exploratory observational cohort study received approval from the ethics commission of the Westphalian Medical Board (approval number: 2019-147-f-S) and was conducted in accordance with the Declaration of Helsinki. Participants provided informed consent prior to their participation in this study. The trial was registered at the German Clinical Trial Register (DRKS00019929).

### Subjects and study protocol

Seventeen adult elite athletes diagnosed with SRC according to the criteria of the Concussion in Sport Group^23^ and 17 healthy, matched controls were included in the study. Matching criteria were sex, age, height, weight, sport, and expertise level (see Table 1). SRC athletes were recruited by actively screening national news articles, reports from (local) sports clubs, and athletes attending the sports neurology clinic at the Institute of Sports Medicine at Paderborn University, Germany. Exclusion criteria for both groups were cardiovascular, mental, or physical disability, diabetes mellitus, and pregnancy. Control subjects were excluded from analysis if they had a concussion within the last year. The medical history assessment encompassed attention-deficit/hyperactivity disorder (ADHD), thyroid dysfunction, learning disabilities, migraines/headaches, and concussion history, including the number of prior concussions (see Table 1).

Data was collected using a multimodal wearable device (Empatica^®^ E4, Milan, Italy) to be worn at home on the non-dominant wrist during all nights of individual RTS. Clinical recovery was defined as the completion of the final stage of the RTS protocol, which equals the return to normal gameplay^23^. Four concussed athletes reached this stage at a time when no competitions took place (COVID-19 and/or off-season) but were still fully cleared. After finishing RTS (> 3 weeks, TP2), athletes were asked to conduct one additional nocturnal recording. Matched control subjects were advised to wear the sensor for the same number of nights as their concussed counterparts, except for the night after the completion of RTS. Thus, control athletes were assessed over a single observation period, and longitudinal interference is limited to the SRC groups. Concussion symptoms and symptom severity were assessed with the symptom checklist of the SCAT5^23^ at the time of inclusion into the study.

### Measurement device

The wearable device recorded blood volume pulse (BVP, sampling rate: 64 Hz), EDA (4 Hz), skin temperature (4 Hz), and 3-axis accelerometer data (32 Hz). It has been validated against an electrocardiogram during nocturnal measurements^43^ and has additionally been applied for monitoring EDA during sleep^44^. Subjects were instructed to manually set markers in the evening (upon going to bed) and in the morning (upon awakening) to create timestamps in the raw data signal. Data was saved on the device’s internal memory and downloaded via a computer.

### ANS parameters and analysis

Nocturnal BVP and EDA were cut using a custom-built script (Python, version 3.9.12). Sleep onset was defined as the first 10 min without any movement^45^ after the evening marker. This was visually determined based on accelerometry data. Wake-up time was approximated using the morning marker. To reduce day-to-day variation^18^ the mean of each ANS parameter from the first four recorded nights (TP1) was calculated and analyzed for SRC and control athletes. If one night of data was missing, the next available night during the RTS period was included instead.

The resulting BVP segment was further preprocessed using Kubios HRV Premium (Biosignal Analysis and Medical Imaging Group, Kuopio, Finland; version 3.5.0)^46^. The pulse acceptance threshold was set at 50%. Ectopic beats were identified and corrected using the automatic artifact correction algorithm, interpolating them by adjacent interbeat intervals (IBIs). The software’s automatic noise detection (default setting: medium) was applied to identify and mark distorted IBIs (e.g., due to movement) as “noise segments”^46^. Noise segments were excluded from the analysis. As this automatic noise detection is intentionally conservative, all identified noise segments were subsequently reviewed by a trained investigator (ACDB) to ensure that valid physiological data were not unnecessarily excluded. Data sets with more than 25% noise segments (effective data length < 75%) or with over 10% corrected IBIs were excluded from further analysis^47^. The early sleep phases mostly contain deep sleep, minimizing the influence of body movements and external factors on ANS measurements^42,48^. Therefore, nocturnal parasympathetic activity (RMSSD) and HR were assessed by calculating the mean over the first 4-h period following sleep onset. A moving window function (window width: 5 min, shift: 1 min) was applied, consistent with established methodological recommendations^49^ and previously published studies^50,51^.

The EDA segment was detrended, low-pass-filtered (Butterworth filter: 4th order, cut of frequency: 0.4 Hz), and smoothed (factor 9)^52^ within the custom-built script. Artifacts were visually inspected and replaced by interpolation. All visual inspections were performed by a trained investigator (ACDB) using standardized criteria. EDA indicates the sympathetic activity of eccrine sweat glands^9^ and outcomes are divided into a tonic component, characterized by a constantly, slowly varying baseline, and a phasic component, characterized as rapid, situational adaptations to internal and external stressors (EDRs): rise > 0,02 µS/s)^53^. Tonic and phasic components were analyzed separately. Continuous decomposition analysis (CDA) in LedaLab (version 3.4.9)^54^ was applied to extract tonic (CDA.Tonic) and phasic (CDA.SCR) EDA over the first 4-h period of the night, in accordance with the HRV analysis. Additionally, prior research has shown that EDRs are most frequently observed during the first half of the night, when deep sleep predominates^41^. However, sleep stage distribution was not assessed in this study. The number of sympathetic sleep storms as nocturnal “surges” of EDA (min. 3 EDR/30 s^39^ over the whole night were determined by the custom-built script, as LedaLab cannot calculate this parameter.

### Statistical analysis

Data were analyzed using SPSS (version 28, IBM Corporation, Armonk, New York, United States). Normal distribution was checked using the Shapiro-Wilk test. Differences between SRC groups (rRTS and pRTS) and controls in demographics, concussion symptoms, and nocturnal ANS data were evaluated using the Kruskal-Wallis Test. Post-hoc tests (Dunn-Bonferroni-Tests) and correction for multiple comparisons using Bonferroni corrections were performed. These analyses were considered confirmatory, as they directly addressed the primary study objectives.

To account for interindividual differences in ANS activity during the RTS phase, exploratory sensitivity analyses were performed using analyses of covariance (ANCOVA). Post RTS (TP2) ANS measures were compared between athletes with regular and prolonged RTS, with the corresponding during RTS (TP1) values entered as covariates. Given the small sample size and exploratory nature of the study, ANCOVA was restricted to a single-covariate model to reduce the risk of model overfitting.

The medical history (pre-existing conditions) was compared between SRC groups and controls using Fisher’s Exact Test, which is appropriate for categorical data with small sample sizes. The percentage change of ANS parameters (TP1 vs. TP2) was calculated in SRC athletes. Comparisons between the SRC groups (regular vs. prolonged) were conducted with t-Tests or Mann-Whitney U Tests, depending on the normality distribution.

The effect size Cohen’s *d* was computed for normally distributed data, Pearson’s *r* for non-normally distributed data, and the phi-coefficient for the result of Fisher’s Exact Test. The level of significance was set at *p* ≤ 0.05 (2-tailed) a priori.

## Supplementary Information

Below is the link to the electronic supplementary material.

Supplementary Material 1

Supplementary Material 2

## Data Availability

The datasets generated and/or analysed during the current study are not publicly available due to the potential risk of de-identification, as the dataset includes elite-level athletes, but are available from the corresponding author on reasonable request.
