# Supplementary figures and images for: Nocturnal autonomic activity in athletes with regular versus prolonged return to sport after sport-related concussion
Source: Sci Rep. 2026 Mar 26;16:10483. doi: 10.1038/s41598-026-43546-0 (PMC13031713; doi:10.1038/s41598-026-43546-0)

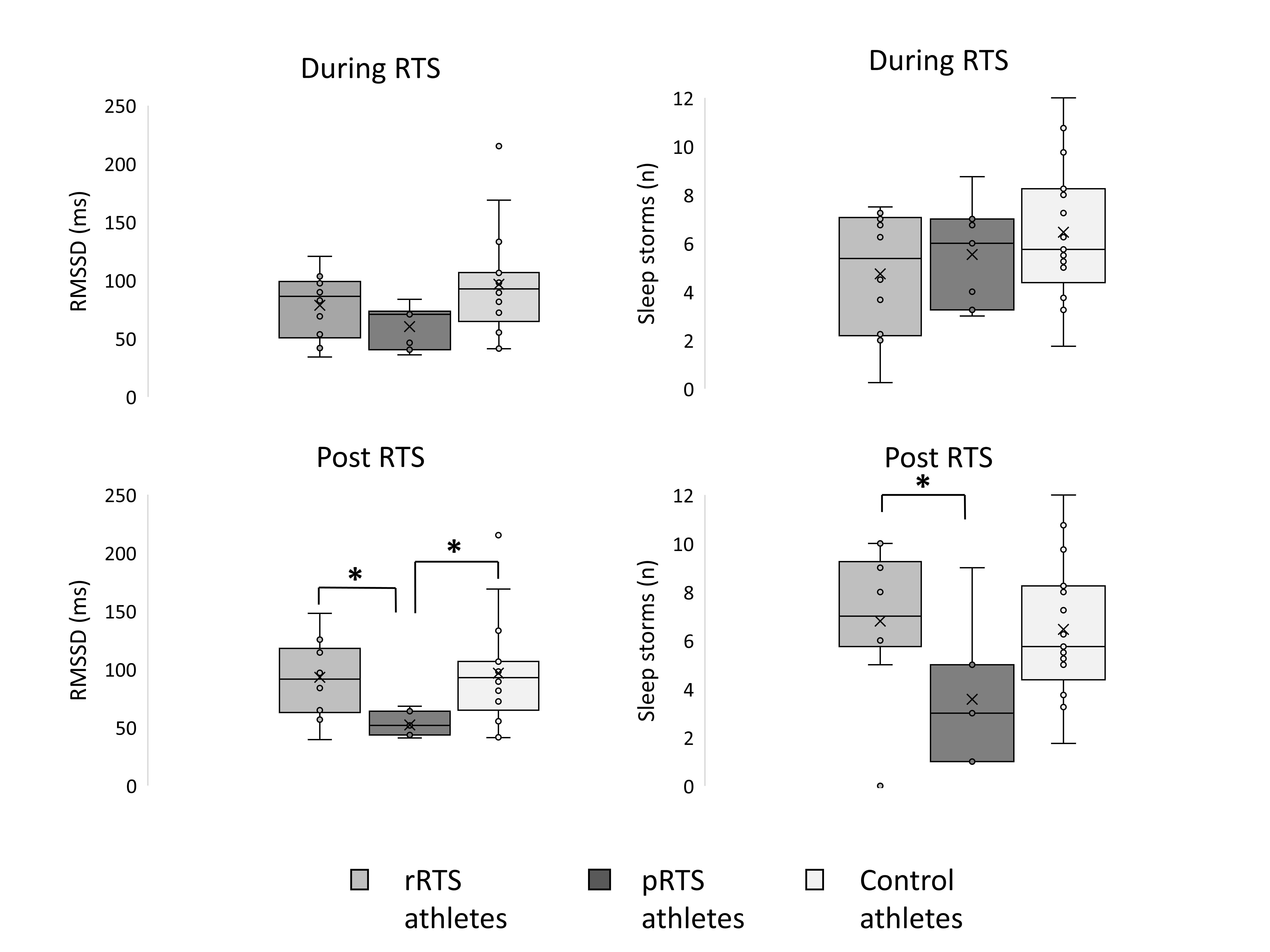

Supplement: Supplementary file 1 — Supplementary Material 1 [file 41598_2026_43546_MOESM1_ESM.tif]
