# Supplementary material for: Nocturnal autonomic activity in athletes with regular versus prolonged return to sport after sport-related concussion
Source: Sci Rep. 2026 Mar 26;16:10483. doi: 10.1038/s41598-026-43546-0 (PMC13031713; doi:10.1038/s41598-026-43546-0)
Supplement: Supplementary file 2 — Supplementary Material 2 [file 41598_2026_43546_MOESM2_ESM.pdf]

Nocturnal autonomic activity in athletes with regular versus prolonged return to sport after sport-related concussion

Anne Carina Delling-Brett[1]; Rasmus Jakobsmeyer[1]; Jessica Coenen[1]; Claus Reinsberger[1,2]*

[1]Institute of Sports Medicine, Department of Exercise and Health, Faculty of Science, Paderborn University, Paderborn, Germany

[2] Division of Sports Neurology and Neurosciences, Department of Neurology, Mass General Brigham, Harvard Medical School, Boston, MA, USA

Supplementary Material

Table S1 Clustering of concussion symptoms in symptom domains, presented as median (± SD)

Symptom domain	Controls (n = 17)	rRTS athletes (n = 10)	pRTS athletes (n = 7)
Somatic	0.00 (± 1.88)	5.50* (± 6.76)	10.00* (± 7.74)
Cognitive	0.00 (± 1.93)	4.00* (± 5.78)	9.00* (± 5.00)
Fatigue/Sleep	0.00 (± 1.78)	3.00 (± 3.65)	5.00* (± 3.15)
Emotional	0.00 (± 1.46)	0.00 (± 4.13)	1.00 (± 4.55)

RTS = return to sport; rRTS = regular RTS; pRTS = prolonged RTS; * $p < 0.05$ different to controls (Kruskal-Wallis-test with post hoc test)

Table S2 Symptom domain clusters

Symptom domain	Associated symptoms
Somatic	Headache,<br>"Pressure in head",<br>Neck pain,<br>Nausea or vomiting,<br>Dizziness,<br>Blurred vision,<br>Balance problems,<br>Sensitivity to light,<br>Sensitivity to noise
Cognitive	Feeling slowed down,<br>Feeling like "in a fog",<br>"Don't feel right",<br>Difficulty concentrating,<br>Difficulty remembering,<br>Confusion
Fatigue/Sleep	Drowsiness,<br>Fatigue or low energy,<br>Trouble falling asleep
Emotional	More emotional,<br>Irritability,<br>Sadness,<br>Nervous or Anxious

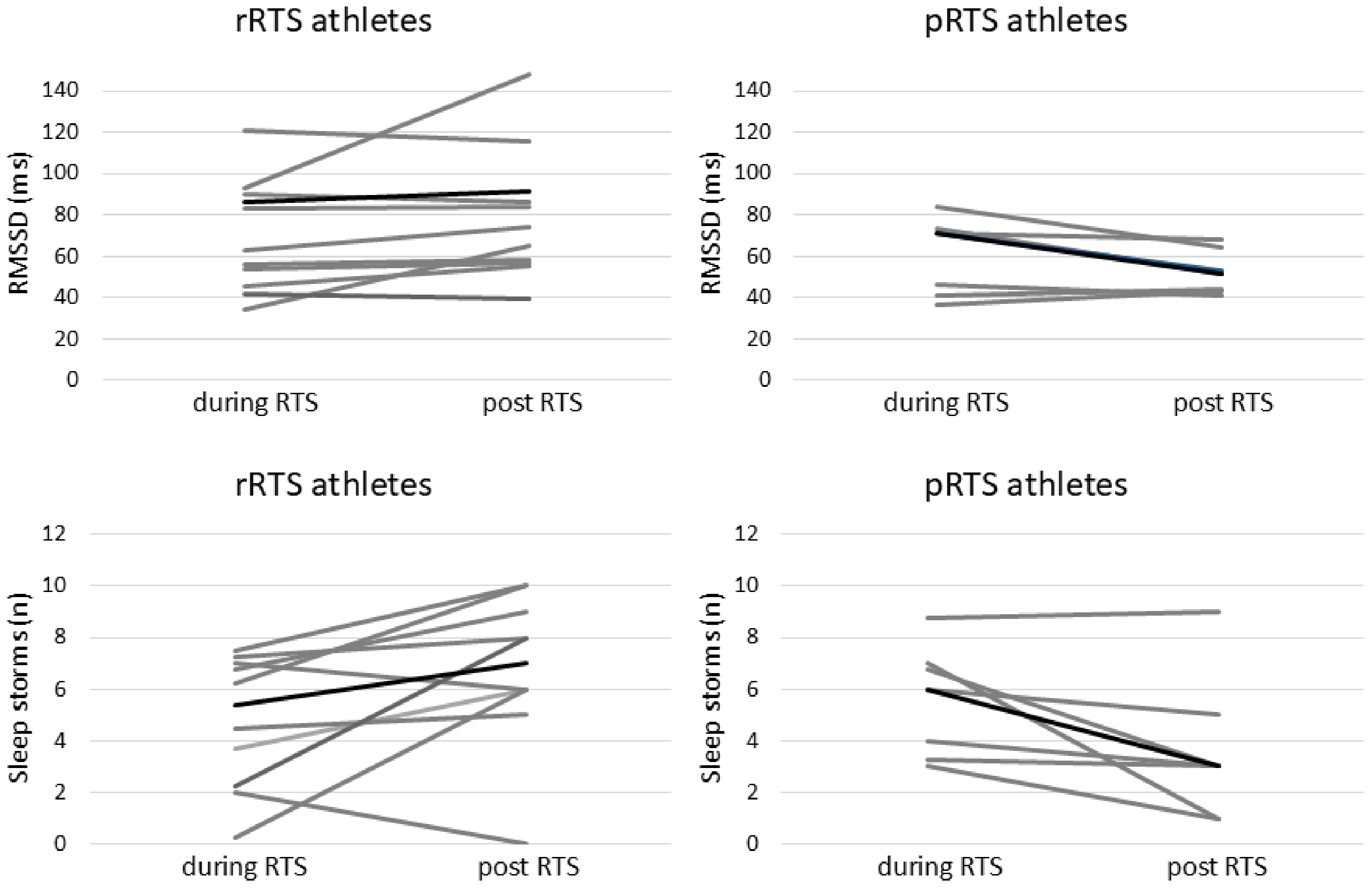

Figure S1 Individual nocturnal RMSSD and sleep storms developments during and post return to sport (RTS) for regular (rRTS) and prolonged (pRTS) RTS athletes. The black line represents the median.
